# Structural basement membrane components and corresponding integrins in Schlemm's canal endothelia

**Published:** 2011-01-19

**Authors:** Saumya S. VanderWyst, Kristin M. Perkumas, A. Thomas Read, Darryl R. Overby, W. Daniel Stamer

**Affiliations:** 1 Biomedical Engineering Graduate Inter-Disciplinary Program, University of Arizona, Tucson, AZ; 2 Department of Ophthalmology and Vision Science, University of Arizona, Tucson, AZ; 3 Ophthalmology, University of Toronto, Toronto, ON, Canada; 4 Bioengineering, Imperial College London, London, United Kingdom

## Abstract

**Purpose:**

The conventional outflow pathway provides the primary source of resistance to aqueous humor drainage, regulating intraocular pressure. Despite large pressure gradients across the inner wall of Schlemm’s canal (SC), cells remain attached to their basement membrane. The goal of this study was to examine integrin-extracellular matrix binding partners of the inner wall basement membrane that facilitate attachment.

**Methods:**

Human outflow tissues and cultured cells were analyzed by immunofluorescence and western blotting, respectively. Radial sections of human donor eyes or en face preparations of human SC inner wall were probed with antibodies that specifically recognize collagens (Type I, III, and IV), laminins (LM-332 and LM-511) and laminin-specific integrin subunits, α3, α6, β1, and β4, typical of vascular endothelia.

**Results:**

Immunofluorescence studies showed collagens Type I and IV in the SC basement membrane but not collagen III. As expected with mature vascular endothelia, SC cells in situ expressed LM-511 but not LM-332. Significantly, the integrin α6 subunit was expressed uniquely by SC. En face labeling of the inner wall displayed integrin α6 colocalizing with LM α5 at the cell periphery. Western blots of cultured human SC endothelial cell monolayers confirmed expression of Type I collagen, collagen IV, LM-511, and the α6 integrin subunit. Interestingly, LM-332 was present in cultured SC cells up to 60 days post-confluence.

**Conclusions:**

Even though cells of the inner wall endure pressure gradients in the basal to apical direction, opposite of other endothelia, human SC cells express basement membrane proteins and their cognate integrins typical of vascular endothelia.

## Introduction

Elevated intraocular pressure (IOP) is a major risk factor for the development of primary open-angle glaucoma (POAG) [1]. In POAG, conventional outflow tissue dysfunction causes increased resistance to aqueous humor drainage, resulting in elevated IOP [2,3]. The principal contributor to increased resistance in the conventional outflow pathway remains unknown; however the location is likely in the juxtacanalicular region of the trabecular meshwork, near the inner wall of Schlemm's canal (SC) [3-9].

With its unique architecture and basal to apical direction of flow, Schlemm's canal inner wall creates a dynamic microenvironment for SC endothelial cells. Massive mechanical loads and a discontinuous basement membrane suggest the necessity of strong cell-matrix adhesion in maintaining a continuous barrier to fluid flow [10,11]. Since the strength of cellular adhesion is dependent on both structural extracellular matrix (ECM) and integrins, it is important to identify and understand their relationship at the inner wall of Schlemm’s canal.

Mature vascular endothelial basement membranes contain self-assembling lattices of collagen IV and laminin-511 connected by molecules of perlecan and nidogen [12]. Additional structural proteins, such as tensile collagen I and other laminins, are also incorporated into the basal lamina [13]. The laminin expression profile of endothelia varies during development and determines tissue function [14]. Endothelial cell integrins also play a large role in maintaining cell-matrix adhesion. Vascular endothelial cells express integrins α3β1, α6β1, and α6β4 to bind to laminin, while collagen-specific integrins include α1β1 and α2β1 [15]. Since SC inner wall endothelia share similarities with vascular endothelia, perhaps SC cells express a similar pattern of basement membrane and integrin proteins [16-18].

Previous attempts to identify extracellular matrix proteins and integrins in Schlemm's canal basal lamina have provided preliminary information [19-25]. However, some studies used antibodies later shown to be non-specific. For example, antibodies previously thought to identify laminin-111 also bind laminin-511 and laminin-411. Recent work with collagens and laminins has resulted in well characterized and selective antibodies for cell-matrix adhesion research.

In the present study, by examining the expression of integrins, collagens and laminins in situ and in vitro, we describe the major structural proteins of Schlemm's canal basal lamina, identify a uniquely expressed integrin subunit by SC cells in the outflow pathway and define variations in ECM expression caused by in vitro culture models.

## Methods

### Human tissue

Post mortem enucleated eyes were obtained from National Disease Research Interchange (NDRI; Philadelphia, PA), the Life Legacy Foundation (Tucson, AZ) and Sun Health Research Institute (Sun City, AZ), according to the guidelines of the Declaration of Helsinki for research involving human tissue. All tissue used was free of known ocular disease and maintained in a humid chamber at 4 °C until dissection.

### SC cell culture

Human SC cells were isolated from donor eyes using a cannulation technique developed in our laboratory and cultured as previously described [26]. The cells were maintained as stable monolayers in Dulbecco’s modified Eagle’s medium (DMEM, low glucose; Invitrogen, Austin, TX), supplemented with 10% fetal bovine serum, penicillin (100 U/ml), streptomycin (0.1 mg/ml) and glutamine (0.29 mg/ml; Life Technologies). Cells were used between passages 2–5 and maintained at confluence for at least one week before testing. Five different primary cell culture strains (SC45, SC51, SC53, SC55, and SC56) were isolated from eye donors (64, 66, 44, 29, and 29 years old, respectively) and were used in experiments as available.

### TM cell culture

Human TM cells were also isolated from donor eyes, using a blunt dissection technique followed by extracellular matrix digestion that was developed in our laboratory [27]. The cells were brought to and maintained at a stable monolayer using Dulbecco’s modified Eagle’s medium (DMEM, low glucose), containing 10% fetal bovine serum, penicillin (100 U/ml), streptomycin (0.1 mg/ml) and glutamine (0.29 mg/ml). Upon reaching confluence, the fetal bovine serum concentration was reduced to 1% and maintained for at least a week until testing. Four different primary cell culture strains (TM89, TM90, TM92, and TM93) were isolated from donor eyes (64 years, 4 months, 38 years, and 35 years old, respectively) and were used in experiments as available.

### Western blot

Media was removed from confluent monolayers of SC and TM cells and rinsed with ice-cold phosphate-buffered saline (PBS; Life Technologies). Cells were mechanically scraped into ice-cold radio-immunoprecipitation assay (RIPA) buffer (Sigma, St. Louis, MO) with protease inhibitors (Complete Mini; Roche, Indianapolis, IN), and chilled on ice for 10 min. The samples were then centrifuged at 20,000× g for 8 min and the supernatant removed, tested for total protein (BCA assay; Pierce, Rockford, IL) and frozen at −80 °C. Lysates were solubilized in Laemmli buffer or non-reducing buffer and boiled for 10 min before fractionating proteins (10 μg total protein/well) on SDS–PAGE gels (4%–15% gradient, 7.5% or 10% non-gradient). Fractionated proteins were transferred overnight onto nitrocellulose membranes by electrophoresis at 21 V. The membranes were blocked with 5% non-fat dry milk in Tris-buffered saline with 0.2% Tween-20 (TBS-T) and probed with antibodies for western blot analysis (see Table 1 for a list of antibody conditions). After being rinsed four times for 10 min in TBS-T, HRP-conjugated secondary antibodies (Jackson Immunoresearch, West Grove, PA) were added. Chemiluminescent substrate (HyGLO, Denville or ADVANCE; Pierce) was added to the membranes immediately before exposure to X-ray film (Genesee, San Diego, CA).

**Table 1:** Antibodies used for western blotting.

**Antibody**	**Dilution**	**Clone/catalog**	**Gel conditions**
Collagen I	1:5,000	Anti-CN^1^	LB
Collagen IV	1:1,000	IV-4H12^2^	NRB
Laminin α3	1:1,000	BM165^6^	LB
Laminin α5	1:1,000	sc-20145^4^	LB
Laminin β3	1:1,000	sc-5583^4^	LB
Laminin γ1	1:500	Clone 17^5^	LB
Laminin γ2	1:1,000	2E8^3^	LB
Integrin α1	1:500	FB12^3^	NRB
Integrin α2	1:1,000	AB1936^3^	LB
Integrin α3/α6	1:10,000	AA6A^7^	NRB

^1^Immunohistochemical research (Gilbertsville, PA). ^2^Calbiochem (San Diego, CA). ^3^Millipore (Billerica, MA). ^4^Santa Cruz Biotechnologies (Santa Cruz, CA). ^5^BD Biosciences (Franklin Lakes, NJ). ^6^BM165; Dr. Robert Burgeson, Harvard University, Cambridge, MA and Dr. Peter Marinkovich, Stanford University, Palo Alto, CA, ^7^Clone 4C7; Millipore, Billerica, MA. In the table, NRB indicates non-reducing buffer and LB indicates Laemmli buffer.

### Confocal immunofluorescence microscopy

Radial wedges excised from the anterior segments of fresh human post mortem eyes were frozen in optimal cutting temperature compound (OCT; Sakura, Torrance, CA). The tissue was sectioned (6.5 μm) radially and fixed for 10 min in ice-cold acetone. The slides were incubated for 30 min in the following antibodies diluted in PBS: anti-collagen Type I (Biogenesis/AbD Serotec, Raleigh, NC), anti-collagen III (Biogenesis Labs and Abcam, Cambridge, MA), anti-collagen IV alpha 1 (Clone H11; gift of Dr. Yoshikazu Sado, Shigei Medical Research Institute, Japan), anti-integrin alpha 6 (JIB5; Caroline Damsky, Department of Cell and Tissue Biology, UC San Francisco, San Francisco, CA), anti-integrin alpha 3 (P1B5; Millipore, Billerica, MA), anti-integrin beta 1 (P4G11, Chemicon), anti-integrin beta 4 (Chemicon), anti-laminin alpha 3 (BM165; Dr. Robert Burgeson, Harvard University, Cambridge, MA and Dr. Peter Marinkovich, Stanford University, Palo Alto, CA), anti-laminin alpha 4 (FC10; generated by Dr. Artturi Virtanen, University of Helsinki, Finland), or anti-laminin alpha 5 (Clone 4C7; Millipore). The slides were then rinsed three times for 5 min in PBS before incubation with secondary antibodies (Alexa-Fluor 488 goat anti-mouse, goat anti-rabbit, or goat anti-rat) for 30 min. The slides were washed once more and then mounted and viewed with a confocal microscope (Zeiss 7700L, Carl Zeiss, Inc, Thornwood, NY). We evaluated background labeling and tissue autofluorescence by incubation of tissue sections with secondary antibodies alone and imaging under identical conditions as the experimental samples.

### Immunoprecipitation

Confluent monolayers of SC or TM cells were rinsed in ice-cold PBS and then mechanically scraped into ice-cold RIPA buffer and centrifuged, as described before. In parallel, protein G beads (Immunopure; Pierce) were washed with RIPA buffer. Solubilized cell supernatants were pre-cleared with an aliquot of washed beads for 1 h at 4 °C with rotation. Aliquots of cell supernatants (100 μg total protein) were used for each immunoprecipitation. Antibodies (1 μg) were added to each samples and rotated for 1 h at 4 °C. Upon addition of the beads, the sample + antibody + bead solution was rotated at 4 °C overnight. Antibody complexes were isolated by centrifugation at 6,000× g for 1 min. After removing supernatant, the beads were washed three times (500 μl) with ice-cold RIPA buffer before loading buffer (40 μl) was added to the beads. After boiling at 100 °C for 10 min and centrifugation at 6,000× g for one min, the supernatants were analyzed by SDS–PAGE and western blotting as described before. Reverse co-immunoprecipitations for laminin-511 were performed to determine whether laminin subunits assembled. The antibody pair used was laminin α5 (sc-20145) and laminin γ1 (2E8). In addition, immunoprecipitation to specifically detect integrin α6 in SC and TM cells used integrin α6 antibody (J1B5) as the pull-down antibody and integrin α3/α6 antibody (AA6A) to probe.

### En face labeling

Human eyes were obtained from the Eye Bank of Canada (Ontario Division, Toronto, Canada) and stored in moistened chambers at 4 °C before use. To preserve outflow structures, eyes were perfused using standard methods [28] and fixed under pressure (8 mmHg) by anterior chamber exchange with 3% formalin in PBS and perfused for a further 30 min. They were then removed from the system, hemisected and the outflow tissue cut into wedges and stored in 15% glycerol in PBS at −20 °C. Radial segments of the limbal area were microdissected using a modification of previously described techniques [29,30]. Briefly, the TM and adherent inner wall of SC were carefully folded anteriorly. This procedure opened SC like a book (with the book spine analogous to the anterior margin of the canal) allowing en-face visualization of both the inner and outer wall endothelia. Tissue was permeabilized in cold acetone for 10 min and blocked with 10% goat serum (Sigma)/10% donkey serum (Jackson Immunoresearch) in PBS, for 30 min at room temperature. The inner wall and underlying TM were labeled with antibodies to visualize laminin α5, integrin α6, CD31 and nuclei. Laminin α5 was labeled with rabbit anti-Laminin α5 IgG (Santa Cruz Biotechnology, Santa Cruz, CA) diluted 1:50 in PBS and incubated overnight at 4 ⁰C, followed by incubation in Alexa-647 goat anti-rabbit IgG (Invitrogen, Austin, TX) diluted 1:150 for 3 h. Integrin α6 was labeled with rat anti-Integrin α6 (gift of Damsky Lab) diluted 1:50 in dPBS and incubated overnight at 4 °C, followed by incubation in Cy3 goat anti-rat IgG (Abcam, Cambridge, MA) diluted 1:100 in dPBS, for 3 h at RT. CD31 was labeled with mouse anti-CD31 (DAKO, Glostrup, Denmark) in dPBS and incubated overnight at 4 °C, followed by incubation in Cy2 donkey anti-mouse IgG (Jackson Immunoresearch), diluted 1:100, for 3 h at RT. For negative controls, tissue was treated as above while excluding the primary antibodies. Nuclei were stained by incubation for 5 min at RT in DAPI (Invitrogen, Austin, TX) diluted at 2 µg/ml in PBS. Samples were examined using a Zeiss LSM 510 meta confocal microscope (Carl Zeiss Inc., Jena, Germany). Images were viewed using Zeiss LSM Image Browser version 4.2 (Carl Zeiss Inc.).

## Results

**Figure 1:**
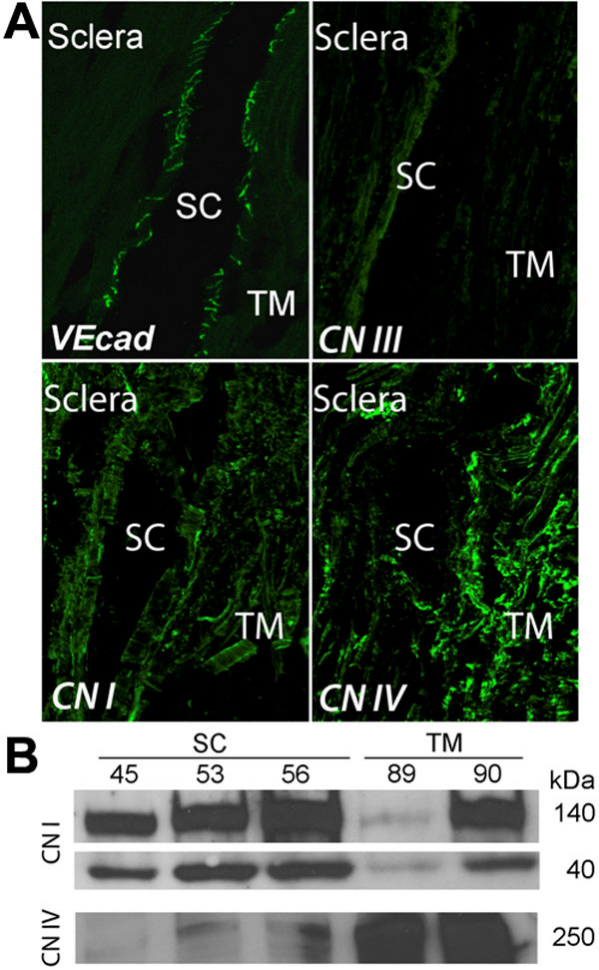
Collagens in human conventional outflow pathway and cultured cells. **A**: Confocal immunofluorescence microscopy of outflow tissues from cadaveric human eyes examining the expression levels of collagen (CN) I, III, and IV. Vascular endothelial cadherin (VE-cad) labeling was used as positive control for integrity of tissue antigens and localization of Schlemm’s canal (SC). Shown are representative images taken from section of one donor eye of 3 examined. **B**: Western blot analysis of collagen levels in different strains of cultured human trabecular meshwork (TM) and SC cell monolayers isolated from different individual eye donors. Anti-collagen I IgGs recognized multiple bands between 140 and 40 kDa, corresponding to post-translational modifications.

To better understand the mechanisms by which cells of Schlemm’s canal (SC) inner wall withstand pressure gradients in the basal to apical direction and remain adherent to their basement membrane, we examined expression levels of key matrix and adhesion proteins by Schlemm’s canal endothelia (vascular endothelial cadherin labeling marks SC; Figure 1A). We found that a primary structural component of the extracellular matrix, type I collagen, was a prominent component of SC inner wall basement membrane in situ as well as in the TM (Figure 1A). Similarly, we observed collagen IV, present in all basement membranes in the conventional outflow pathway, including SC’s inner wall (Figure 1A). We were unable to detect type III collagen in outflow pathway tissues, but did see positive staining in human crystalline lens capsule (data not shown). Both type I and type IV collagen were produced by mature monolayers of SC and TM cells in culture. Type I collagen in vitro was represented by multiple bands due to post-translational modifications and protein solubility (Figure 1B). While the production of collagen I was similar between SC and TM cells, collagen IV production was much greater in TM cells.

**Figure 2:**
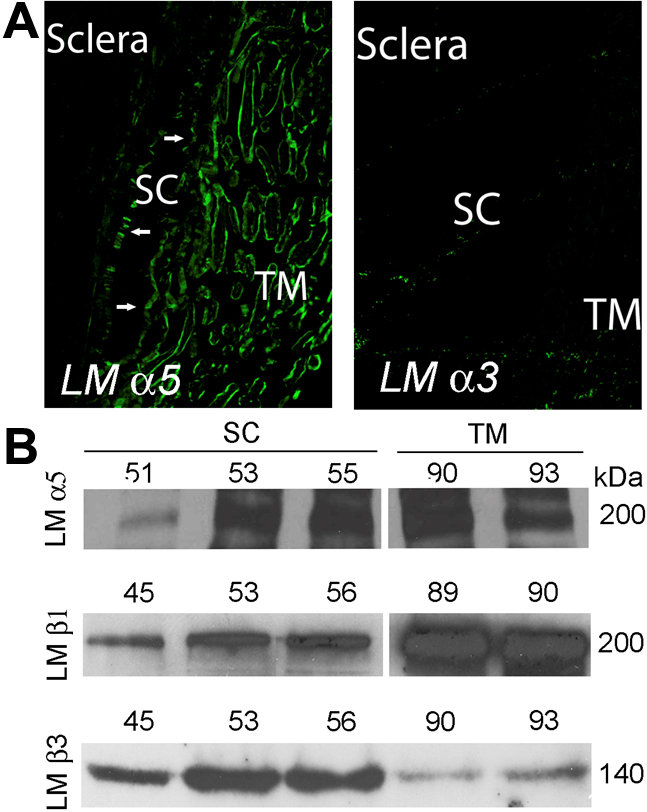
Laminins-332 and −511 in human conventional outflow pathway and cultured cells. **A**: Confocal immunofluorescence microscopy of endothelial laminins (LM) in radial sections through human conventional outflow tissues. Shown are representative images taken from one human donor eye of three that were examined. **B**: western blot analysis of endothelial laminin levels by different strains of cultured SC and TM cell monolayers isolated from different individual eye donors. Samples for laminin α5 blots were obtained after cell lysates were first immunoprecipitated with anti-laminin γ1 IgGs.

Laminin-511 is the dominant laminin type expressed by both SC, including at the inner wall, and TM tissues in situ (α5; Figure 2A). In contrast, we were unable to detect laminin-332 in the conventional tract (α3; Figure 2A). Positive staining with laminin-332 antibodies was observed in human prostate tissue (data not shown). We looked at these two laminins in vitro and confirmed that cultured SC and TM cell monolayers produced laminin-511 (α5 and β1). To verify that subunits were being assembled into heterotrimers, co-immunoprecipitation assays were executed, pulling down protein complexes with antibodies that recognize the γ1 laminin subunit and probing blots with anti-α5 antibodies (Figure 2B). Interestingly, we observed that both outflow cell types also produce laminin-332 (β3; Figure 2B) with SC producing noticeably more even in cell monolayers confluent for 8 weeks.

**Figure 3:**
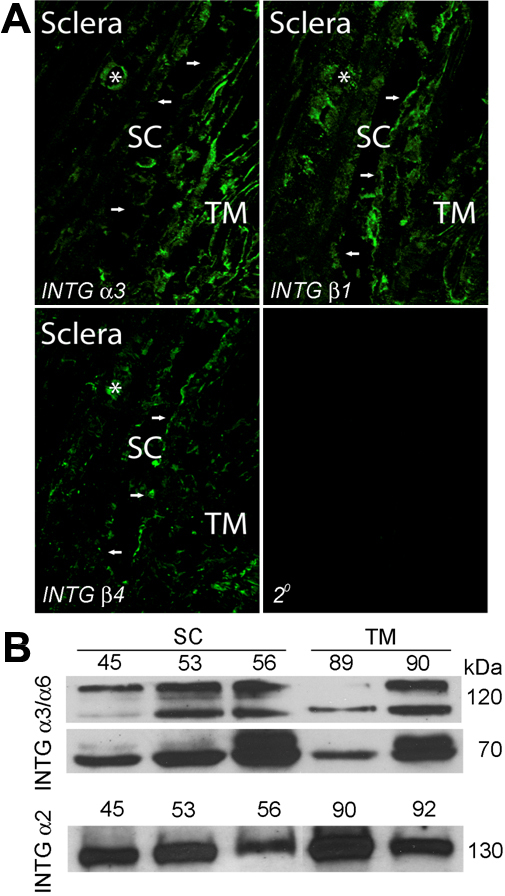
Vascular endothelial integrins in human conventional outflow pathway and cultured cells. **A**: Confocal immunofluorescence microscopy of outflow tissues from human, post-mortem eyes examining integrin (INTG) subunit levels. Shown are results obtained from one eye from one individual donor of three total that were examined. Background fluorescence in the absence of primary antibodies is shown (2°). **B**: western blot analysis of integrin levels by different human Schlemm’s canal (SC) and trabecular meshwork (TM) cell strains in culture.

**Figure 4:**
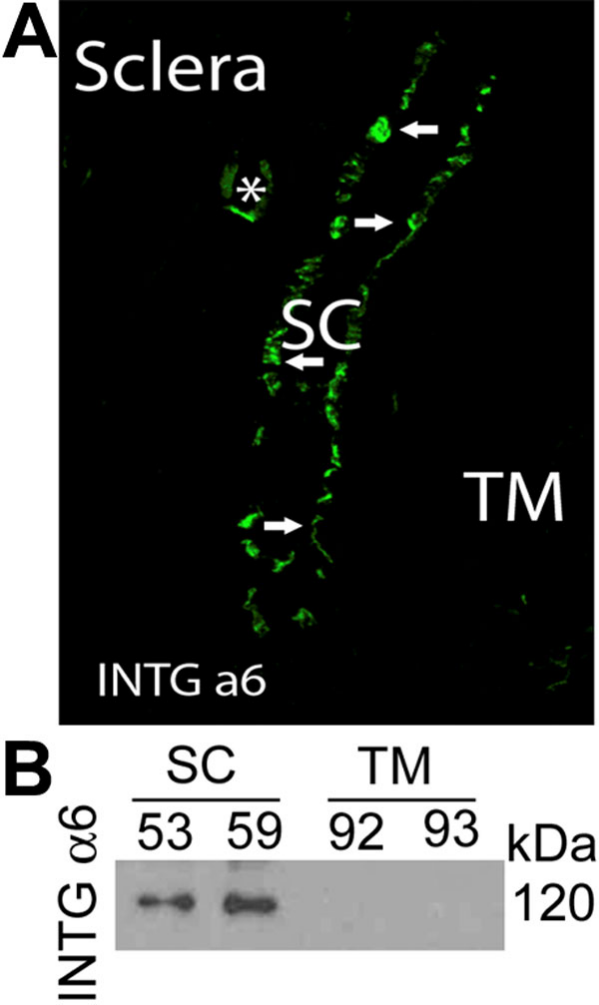
Integrin α6 subunit expression by Schlemm’s canal cells. **A**: Confocal immunofluorescence microscopy of outflow tissues including Schlemm’s canal (SC) from human donor eyes showing α6 integrin (INTG) levels. Shown is representative image from eye of individual human donor of 3 donor eyes that were examined. For comparison, a scleral vessel is indicated (*). **B**: western blot analysis of different human SC and trabecular meshwork (TM) cell strains checking for α6 integrin levels. Cell lysates were first subjected to immunoprecipition using integrin α6-specific IgGs, followed by SDS–PAGE and western blotting using IgGs that recognize both α3 and α6 INTG.

To investigate the integrin pairs, namely α1β1, α2β1, α3β1, α6β1 and α6β4, that may be mediating adhesion of SC to its basal lamina, we examined human tissue and cells for expression levels of individual integrin subunits. The promiscuous integrin-β1 and collagen-specific integrin-α2 were abundantly expressed by both TM and SC cells in situ (Figure 3A) and in vitro (Figure 3B), respectively. With respect to laminin-specific subunits, we found α3, α6, and β4 in conventional outflow pathway tissues (Figure 3A and Figure 4A). Expression was confirmed for integrin α3/α6 in cultured cell monolayers, showing similar expression levels between SC and TM cells (Figure 3B). In contrast, we observed that integrin α6 was uniquely expressed by Schlemm’s canal in the outflow tract in situ (Figure 4A) and by scleral blood vessels. We confirmed the restricted expression pattern of integrin α6 in cultured SC cell monolayers (Figure 4B), not detecting expression by TM cells.

**Figure 5:**
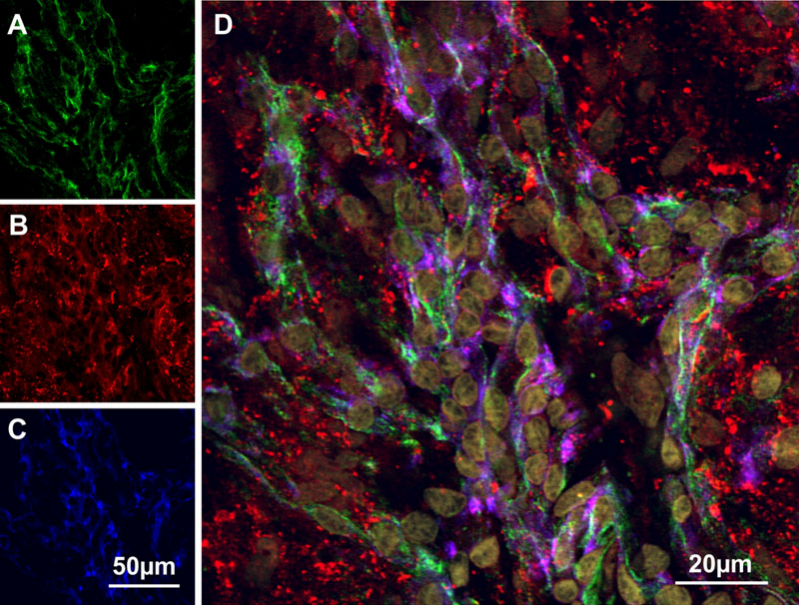
En face confocal immunofluorescence microscopy of Schlemm’s canal inner wall. Shown is labeling of the inner wall of SC (viewed from luminal side) for integrin α6 (**A**, green), laminin α5 (**B**, red), CD31 (**C**, blue). Panel **D** is a merged image of all three proteins plus nuclei (brown). Shown is representative image from a human donor eye of 6 total eyes that were examined.

To examine the relationship between integrin α6 and laminin α5, we performed en face confocal imaging of the inner wall of SC that was probed with antibodies specific for integrin α6, laminin α5 and CD31. We used the endothelial-specific marker, CD31, to locate SC endothelial cells and simultaneously captured fluorescent signals from the other two proteins. We observed that integrin α6 displayed a perinuclear distribution that extended to cell borders (Figure 5A). Laminin α5 appeared as a distinct pattern of labeling over the field of view with openings that roughly corresponded to cell nuclei (Figure 5B). The merged image (Figure 5D) shows that integrin α6 co-localized with CD31 on the cell surface, but extended further to the cell periphery, where laminin α5 and integrin α6 appeared to colocalize.

## Discussion

In the present study we examined the expression of collagens, laminins and their cognant integrin receptors, describing the major structural proteins of human Schlemm's canal (SC) basal lamina in situ and in cultured human SC monolayers in vitro. We identified collagen I, IV, and laminin-511 as prominent structural proteins in the SC basal lamina plus the integrin subunits, α2, α3, α6, β1, and β4. Significantly, we found that α6 was uniquely expressed by SC cells in situ in the conventional outflow tract and in vitro by cultured SC cells, representing a novel cell marker. Moreover, in colocalization studies we observed that laminin-511 and integrin α6 expression overlapped at the cell periphery of SC, coincident with regions of the inner wall displaying high adhesive strength in vivo.

### Collagens

With a triple helix made of post-translationally modified monomers, type I collagen fibrils are able to extend indefinitely and provide tremendous tensile strength. Numerous studies have established the presence of collagen I in ocular tissues, including SC and TM [31]. Our in situ results are similar to results found previously [19,21,25,32-37] and are supported for the first time by in vitro data with cultured human SC cell monolayers. Another collagen in vascular basal lamina is collagen IV, a basement membrane-specific lattice collagen [38]. The stability of the collagen IV lattice provides considerable structural integrity to the basal lamina and allows the matrix to compensate for mechanical strain [12]. A previous study found collagen IV in outflow tissues by probing at an ultrastructural level [21]. We confirmed collagen IV expression in situ in the basal lamina of Schlemm’s canal and showed that SC cells produce collagen IV in vitro, but not to the same extent as TM cells. The abundant expression of collagen IV by TM cells in culture may be reflective of their role in vivo in maintaining an extensive network of lamellar beams in the conventional outflow tract [32,33] or may be a function of cell culture conditions.

### Laminins

Laminin proteins are currently known by the shorthand “LM” followed by a numeric corresponding to the alpha, beta, and gamma chains of the heterotrimeric molecule [39]. For example, laminin-5, also known as kalinin, is referred to as laminin-332 and laminin-10 is called laminin-511. Identifying laminin subunits has gotten better with more specific antibodies. For instance, antibodies previously thought specific for laminin α1, only expressed in laminin-111 (LM-1) and laminin-121 (LM-3), have been found to also recognize laminin α5, present in both laminin-511 and laminin-521 (LM-11). Antibodies currently available, including those used in the present study, are more selective for individual monomers.

Recently, laminin research has undergone a major shift concerning the role of laminin in the ECM and the identification of laminin subunits [15,40,41]. As a required part of basement membranes, laminins have shifted from being viewed as structural scaffolding to being hailed as a determinant in tissue function with various isoforms found to be cell and tissue specific. Moreover, their spatial and temporal specificity has been determined to affect tissue functionality. For example, laminin-332 is expressed by endothelial cells during early development and angiogenesis [42]. Upon establishing a mature vasculature, the expression of laminin subunits in vivo shifts to LM-511 [43]. While integrin-receptor pairs have not been uniquely identified for every laminin heterotrimer, a change from LM-332 to LM-511 expression roughly corresponds to integrin subunit expression shift from integrin α3 to α6 [44]. The observed α6 integrin expression in situ and in vitro by SC suggests that SC cells in culture have begun to differentiate, preparing for a LM-511 containing basement membrane.

Our studies support the premise that laminin expression varies based upon functionality and developmental stage. For example, we show that laminin-511, but not laminin-332 is expressed in situ in human outflow tissues. In contrast, we observed in vitro that both the “immature” (laminin-332) and “mature” (laminin-511) proteins were produced by cultured SC and TM cell monolayers. Even though we differentiate cells in culture for two weeks at confluence, clearly they are still in an angiogenic state based upon their secretion profile. As a control, we tested human umbilical vein endothelial cell monolayers, and observed that they also expressed both laminin-332 and laminin-511 at confluence (data not shown).

### Integrins

Integrin heterodimers are composed of two subunits (α and β), connected by covalent bonds. The integrin heterodimers connect the cell cytoskeleton to the ECM, facilitating signal transduction and affecting cell function [15]. Selectivity of binding to the ECM is dependent upon combination of the two subunits [45]. For example, while β1 is a widely expressed subunit, it may be involved in laminin or collagen binding, depending upon the α subunit with which it complexes. For example integrin α1β1 and α2β1 both interact with collagen polymers, while the integrin heterodimers α3β1 and α6β1 are specific to laminins. Interestingly, integrin α6β1 binds selectively to laminin-511 [44]. In the present study, not surprisingly, we found that both TM and SC cells express the collagen-binding integrin α2 and laminin-binding integrin, α3. In contrast, SC cells both in situ and in vitro uniquely expressed integrin α6. We were fortunate to have a blood vessel present in most of our tissue sections as an internal positive control, confirming the known expression pattern of vascular endothelial integrins (i.e.: α3β1, α6β1, and α6β4) [43,46-48]. Our results are in agreement with the vasculature literature, but different from another study [24] that did not find integrin β4 in SC, likely due to differences in available antibodies.

To examine the potential relationship between integrin α6 (α6β1) and laminin-α5 (511) at the level of the SC basement membrane, we employed en face imaging of the inner wall of SC from human eyes. This technique allows simultaneous capture of fluorescent signals from 4 sources (integrin α6, laminin-α5, CD31 and nuclei) from the luminal surface of an intact inner wall [29,30]. In our studies we observed co-localization between laminin α5 and integrin α6 at the periphery of SC cells. These data are consistent with the idea that this particular integrin-laminin interaction provides adhesive support to secure SC cells to their basement membrane in opposition to basal to apical flow across the endothelia. Significantly, the cell periphery, near sites of cell-cell adhesions are often anchor points for SC cells that have lifted off of their basement membrane, forming “giant vacuoles” in the face of flow. To better understand the role of integrin-laminin adhesion in giant vacuole formation, it would be interesting to examine inner wall regions that experience high flow compared to low flow for differences in localization and/or expression of integrin-laminin pairs in future studies.
